# Polymethyl Methacrylate-Based Bone Cements Containing Carbon Nanotubes and Graphene Oxide: An Overview of Physical, Mechanical, and Biological Properties

**DOI:** 10.3390/polym12071469

**Published:** 2020-06-30

**Authors:** Sanaz Soleymani Eil Bakhtiari, Hamid Reza Bakhsheshi-Rad, Saeed Karbasi, Mohamadreza Tavakoli, Mahmood Razzaghi, Ahmad Fauzi Ismail, Seeram RamaKrishna, Filippo Berto

**Affiliations:** 1 Advanced Materials Research Center, Department of Materials Engineering, Najafabad Branch, Islamic Azad University, Najafabad, Iran; s_sanaz23@yahoo.com (S.S.E.B.); mahmood.razzaghi@gmail.com (M.R.); 2 Biomaterials and Tissue Engineering Department, School of Advanced Technologes in Medicine, Isfahan University of Medical Sciences, Isfahan 81746-73461, Iran; karbasi@med.mui.ac.ir; 3 Department of Materials Engineering, Isfahan University of Technology, Isfahan 84156-83111, Iran; mtavakoli6323@gmail.com; 4 Advanced Membrane Technology Research Center (AMTEC), Universiti Teknologi Malaysia, Skudai, Johor Bahru, Johor 81310, Malaysia; afauzi@utm.my; 5 Department of Mechanical Engineering, National University of Singapore, 9 Engineering Drive 1, Singapore 117576, Singapore; seeram@nus.edu.sg; 6 Department of Mechanical and Industrial Engineering, Norwegian University of Science and Technology, 7491 Trondheim, Norway

**Keywords:** bone cements, carbon, polymethylmethacrylate, reinforcement materials

## Abstract

Every year, millions of people in the world get bone diseases and need orthopedic surgery as one of the most important treatments. Owing to their superior properties, such as acceptable biocompatibility and providing great primary bone fixation with the implant, polymethyl methacrylate (PMMA)-based bone cements (BCs) are among the essential materials as fixation implants in different orthopedic and trauma surgeries. On the other hand, these BCs have some disadvantages, including Lack of bone formation and bioactivity, and low mechanical properties, which can lead to bone cement (BC) failure. Hence, plenty of studies have been concentrating on eliminating BC failures by using different kinds of ceramics and polymers for reinforcement and also by producing composite materials. This review article aims to evaluate mechanical properties, self-setting characteristics, biocompatibility, and bioactivity of the PMMA-based BCs composites containing carbon nanotubes (CNTs), graphene oxide (GO), and carbon-based compounds. In the present study, we compared the effects of CNTs and GO as reinforcement agents in the PMMA-based BCs. Upcoming study on the PMMA-based BCs should concentrate on trialing combinations of these carbon-based reinforcing agents as this might improve beneficial characteristics.

## 1. Introduction

**Figure 1:**
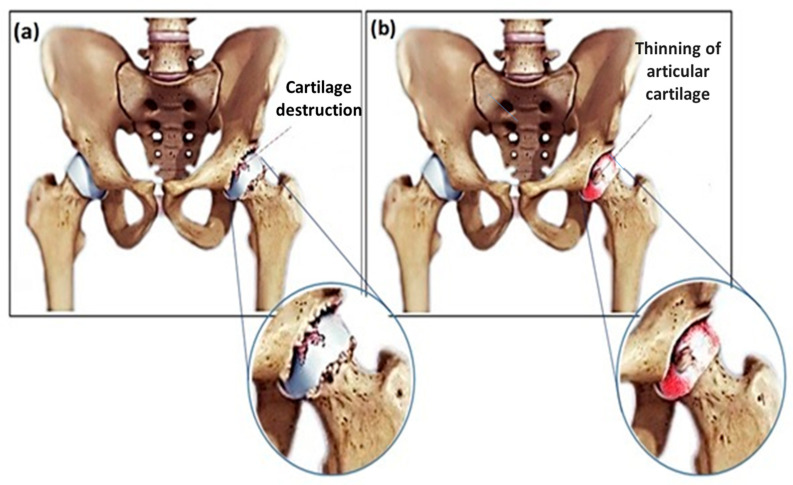
Scheme of (**a**) Hip osteoarthritis and (**b**) Hip rheumatoid arthritis [2].

Diseases like osteoporosis, osteoarthritis, and rheumatoid arthritis can degenerate the joint. Osteoarthritis, a degenerative joint disease that can lead to the breakdown of the cartilage in the joints [1,2], is known as the most typical reason to have a hip or knee replaced as shown in Figure 1 [2]. Total joint replacement (TJR) is a field of treatment for reducing pain and enhancing life quality, and is remarkably used for treating these debilitating illnesses. It is expected that the number of joint replacements is widely increased, due to the escalation of the elderly population as well as associated trauma and disease [1,2]. TJR is known as one of the most usual tasks in orthopedic surgery, considering an appropriate prognosis. The achievement and wide-spreading utilization of joint substitutions in the management of arthritic states along with trauma has caused an essential influence in modern healthcare. The successful results in the cases of total knee/hip joint replacements have provided active lives and also good life quality for us [1,2,3]. In this relation, polymethyl methacrylate (PMMA)-based bone cements (BCs) has been utilized as a fixation agent between the bone and the implant. The PMMA-based BCs are located between the implant and the bone, and the BCs with specific structure can enable the cement for impressive transferring of the body weight and other loads from the prosthesis to the bone [1,2,3,4]. The success of cemented prosthesis is strongly dependent on the reliable interface between the prosthesis and the cement as well as the mechanical bond between the bone and the cement; therefore, a robust bond at the interface between prostheses–cement–bone is essential. Self-curing BCs which can help to anchor the prosthetic parts for contiguous bone are applied in cemented surgery [1,2,3,4]. The main reasons for using BCs in clinical applications are secure processing, excellent bonding capability with other polymers, proper dimensional stability, UV resistance, optical clarity, appropriate chemical resistance, and surface hardness [1,2,3,4]. Addition of biodegradable calcium phosphate (Ca-P) and other carbon-based materials such as carbon nanotubes (CNTs) and graphene oxide (GO) which release no or small amount of heat upon curing have already been produced and their applications in TJR and fractured bone treatments (FBT) have been investigated [4]. In this review paper, we sum up the BCs containing CNTs and GO reinforcements and their composites with other reinforcements in order to enhancement of their clinical applications.

### Historical Background of PMMA- based BCs

PMMA was formulated and introduced as a glass-like solid material with high biocompatibility by Otto Rohm in 1902. In 1936, Kulzer proved that the material with a dough form could be made through the mixing of PMMA powder and a liquid monomer that can be cured when benzoyl peroxide (BPO) is incorporated, and the produced mixing is heated up to 100 °C [3]. The earliest clinical usage of PMMA in 1938 was applied for cranial deficiencies in monkeys. Surgeons turned towards utilizing these materials in the case of reconstructive surgery of humans after obtaining a deeper comprehension of the PMMA system [3]. In this regard, Paladon 65^®^ (Heraeus Kulzer, Hanau, Germany), a type of heat curing polymer, was utilized to repair human cranial defects by taking the advantage of manufacturing plates and trimming the material after curing (in situ) [4]. Polymer chemists found that methyl methacrylate (MMA) polymerization could be accomplished by itself in ambient conditions if a co-initiator is employed, and two companies, Degussa and Kulzer (Heraeus Kulzer, Hanau, Germany) determined an approach for the chemical production of PMMA-based BCs using tertiary aromatic amines. The mentioned procedures have already remained as the key elements for the development of PMMA-based BCs [3]. The first utilization of PMMA for manufacturing a hip prosthesis was done by Judet brothers in 1949 [5]. However, it became certain that the PMMA prosthesis utilized by the researchers may be neither incorporated nor jointed successfully in the body due to mechanical and biological reasons. In 1958, Charnley [6] appropriately stabled an intramedullary stem prosthesis with self-polymerizing PMMA-based BCs and called the materials acrylic-based BCs. He firstly explained a new surgical method of total hip joint replacement surgery in 1970 [7]. In the 1970s, the most feared complexity after total joint substitution [8,9] was the incorporation of antibiotics into BCs for alleviating the periprosthetic infection. The idea was to incorporate antibiotics into the cement for decreasing the occurrence of infections that were numerous at that time. Mixing of gentamicin in powder form with PMMA-based BCs was determined to be firm and presented as an appropriate spectrum of antibiotic activity [10,11].

## 2. Composition of PMMA-based BCs

**Figure 2:**
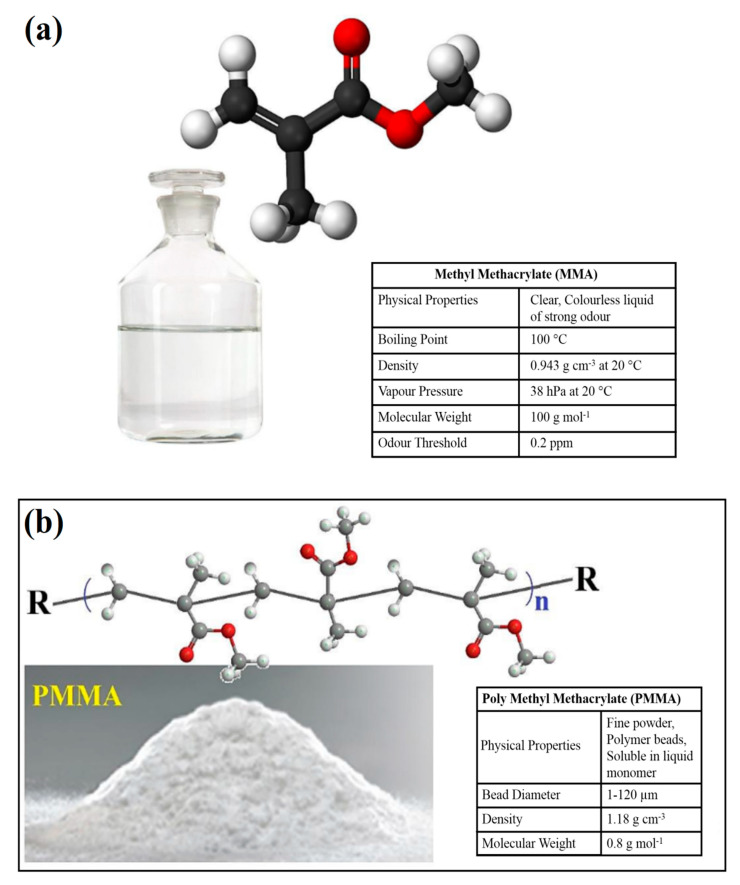
Chemical structures and properties of (**a**) MMA and (**b**) PMMA [11].

PMMA-based BCs are the materials provided with two phases. They consist of a liquid monomer (MMA) and a polymer powder (PMMA) [9,12]. Figure 2 shows the chemical structures and properties of MMA as the liquid phase and PMMA as the polymer powder phase of the PMMA-based BCs [11].

The polymer powder phase consists of PMMA and/or methacrylate copolymers. Moreover, the polymer powder includes BPO that can act as an initiator for the polymerization reaction of the free radicals. Moreover, the powder includes an inorganic radio-pacifying agent, commonly barium sulphate (10–15 wt %) and sometimes an antibiotic [2,3,4]. MMA has been known as the main constituent in the liquid phase; however, sometimes, other methacrylates like butyl methacrylate are provided. For MMA to be employed for BCs it must be polymerizable and the chemical structure which contains carbon double bond should be broken. The liquid consists of an aromatic amine, like *N*,*N*-dimethyl-*p*-toluidine (DmpT) as an activator of the formation of radicals. Besides, for preventing premature polymerization during storage, it includes an inhibitor (hydroquinone) and a coloring agent like chlorophyll as an option [2,3].

### 2.1. Handling Properties of PMMA-Based BCs

**Figure 3:**
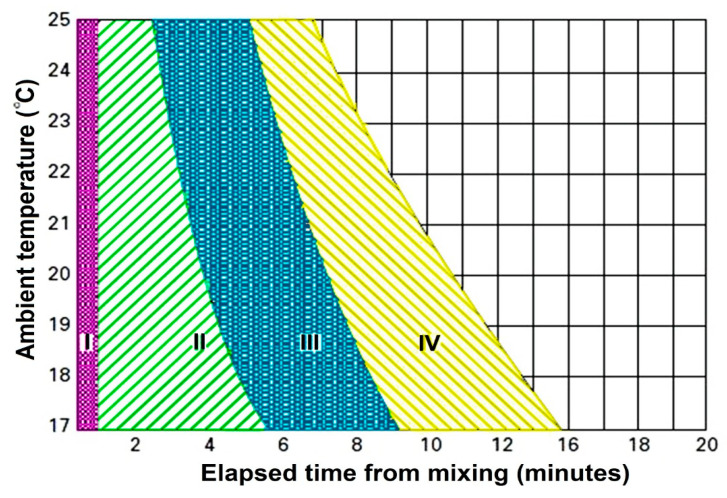
Four polymerization steps for PMMA-based BCs are introduced with four distinct phases that change as a function of the ambient temperature, I: mixing phase; II: waiting phase; III: application phase and IV: setting phase [3].

Figure 3 shows the handling of BCs that is described by four different stages by their relevant viscosities [3]: mixing phase, waiting phase, working or application phase, and adjusting or hardening state [13]. The mixing phase, which takes up to one minute, is the time for perfect homogenization of the polymer powder and the liquid monomer phases. The homogeneity of the dough is affected by using many parameters like the design of the mixing the spatula and vessel, the number of strokes, the mixing speed or revolutions per minute, etc. The more robust and more extended mixing of the bone cement (BC) makes it more porous [9,12,13]. The waiting phase is the period for obtaining a non-sticky consistent cement to be prepared for utilization, and takes up to some minutes based on the handling temperature and the BCs type. The working stage or application is the period in which the BCs is used in the bone before the prosthesis implantation [9,12,13]. It normally takes 2–4 min, which can of course be varied based on the BCs type and temperature of handling. During this phase of polymerization, the cement dough has a mild viscosity. The hardening or setting phase is the final period of this setting process in which the polymerization heat is developed that takes 1 to 2 min. Termination of polymerization can occur when the chain growth of polymer stops. The temperature that is generated by the polymerization can drop back to the ambient temperature at the end of the setting phase [3].

### 2.2. Thermal Properties of PMMA-Based BCs

PMMA polymerization, and thus cement curing, is a highly exothermic reaction. During the exothermic polymerization of BCs, in vivo temperatures may lead to the death of the bone tissue or thermal necrosis and may cause disorder in the circulation of local blood which can cause early failure through aseptic loosening of the implant [9,14]. This reaction releases about 52 KJ per mol of monomer, which results in a heat generation around 1.4–1.7 × 10^8^ J per cubic meter of the cement [15,16]. As a consequence, during its cure, the cement can reach a temperature up to 70–120 °C [13]. It has been demonstrated that thermal necrosis of the bone tissue occurs at the temperatures higher than 50 °C when the exposure exceeds one minute, and that the denaturation of sensory nerves happens at the temperatures higher than 45 °C if the disposal time exceeds 30 min [17]. Consequently, as the cement is in intimate contact with the bone tissue during polymerization, the high temperature reached in the cement may lead to the thermal necrosis of the bone cells and hence lead to essential drawbacks. If this happens, it could compromise the success of the surgery and contribute to the aseptic loosening of the prosthesis [16,18]. Setting properties of BCs are performed based on the ISO5833:2002 and ASTM F451-99a standards [19,20].

### 2.3. Mechanical Properties of PMMA-Based BCs

PMMA-based BCs have a typically mechanical capability by means of which they can equally distribute mechanical stresses and transfer them from the implant to the bone. The mechanical force transfer is obtained using the cement interface, making a considerable surface area, decreasing the stress concentrations over the bone [9,11,12,13]. Charnley proved that the total area that is concealed using the cement surface is about 84 cm^2^, introducing it 65 times more than the interface covered by non-cemented implants [3,7]. In this regard, the mechanical properties of acrylic BCs have been obtained and presented in several studies. However, corresponding to the mechanical properties of BCs, the studies’ results are not comparable due to a lack of information in the case of the storage and preparation of the test samples along with the utilized test methods [21,22,23]. Also, reporting strength characteristics is a challenging task. Cement composition, the molecular mass of the polymer powder phase [3], the use of antibiotics and radiopacifying agents [21,22,23], the porosity [24,25,26], the sterilization approach utilized to the liquid monomer, the polymer powder phases [25], the mixing methods [27,28,29,30], the environmental test conditions [31,32], and the implant design [7,33] are parameters that can affect the mechanical properties and totally mechanical properties of PMMA-based BCs are poor and the key problems of the PMMA-based BCs are mismatch of elastic modulus and stiffness between cement and bone which may cause failure of the BC [28,31,32]. Compressive and bending properties of the PMMA-based BCs are determined in accordance with ISO5833:2002 and ASTM F451-99a standards [19,20].

### 2.4. In Vivo Assessments of PMMA-Based BCs

BCs are materials that are biologically inert, which means that they do not promote the growth and regeneration of the surrounding bone and, consequently, the cement-bone bond is weaker [34]. Several studies have attempted to improve the biocompatibility of the cement, in most cases, by incorporating bioactive agents in the formulation [34] like glass-ceramic particles, glass beads, and hydroxyapatite (HA)-based powders [35,36,37,38]. Based on previous studies [2,16], it is determined that each of these additives can enhance the biocompatibility of BCs and decrease the formation of fibrous tissue at the bone and BCs interface.

## 3. Use of Carbon Compounds in PMMA-Based BCs

**Figure 4:**
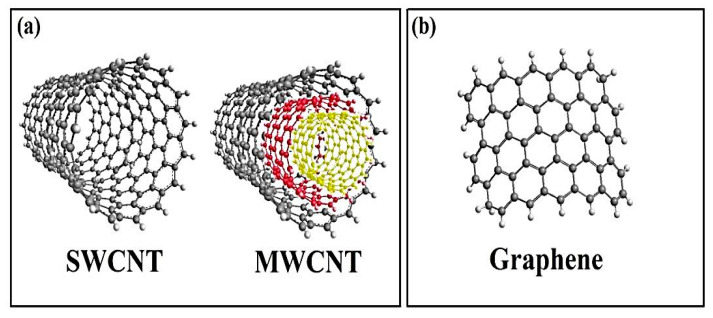
Schematic figure of the morphological structure of: (**a**) CNTs and (**b**) single-layer graphene sheet [58].

Carbon is a light and versatile material and its properties vary extensively depending on the local bonding of the constitution carbon atoms. Some of the most common examples of carbon allotropes classically used in numerous applications are diamond, amorphous carbon, and graphite [16,39]. Carbon-based nano-materials (CBNs) are the most recently discovered allotropes. They could be defined as those carbon allotropes which show one dimension in the nanometer range, providing them with new and special attributes. The most popular ones are CNTs and graphene (G); however, it also includes other ones as GO, nano-plates, fullerenes, or nano-diamonds [16,39]. In 1980, Iijima presented a particle with an onion shape with an approximate diameter of 1 nm [2]. About five years later, he showed that this ‘onion-like structure’ is the fullerene C60 which was believed to be discovered by Kroto, Heath, O’Brien, Curl, and Smalley in 1985, by Kroto in 1985, although [40] later corroborated that Osawa had reported the concept earlier in 1970. In 1991, Iijima showed that the soot deposited over the cathode during the arc-evaporation production of fullerenes caused the observation of a needle-shaped material [41]. Originally, what has been demonstrated as “microtubules of graphitic carbon” is currently introduced as CNT. However, Iijima introduced as the “power of serendipity”. In the first step, Iijima synthesized single graphitic carbon tubes with diameters in the range of 4–30 nm and the length of less than 1 µm by utilizing arc-discharge evaporation techniques [41]. Currently, CNT is produced by electric arc discharge [41,42], laser ablation [43], or, more commonly, chemical vapor deposition [44,45,46]. Extensive research has been done on these approaches in [45] and [47]. CNT occurs as either multi-walled carbon nanotubes (MWCNTs) or single-walled carbon nanotubes (SWCNTs) structures. SWCNTs include a single sheet of graphene that is rolled up as a seamless tube which is considered as a linearly extended fullerene [41,48,49,50]. Commonly, SWCNTs are considered as agglomerations because of the van der Waals forces existent in each tube, with mean diameters in the range of 0.7 to 2 nm; however, their lengths are around 5 to 30 µm [48,51]. MWCNTs can be described as an array of SWCNTs, which are arranged concentrically inside one another with an internal diameter as small as 2.2 nm, concentrically [48,49,50,51]. The existed distance between the individual SWCNTs, which constitute MWCNTs or the graphite inter-layer separation, is usually equal to 0.34 nm [41,48,49]. The ends of CNTs closed off at the attendance of pentagonal carbon rings about the tip areas, whereas imperfections and deformations of the cylinder can happen as heptagons or pentagons in the main structure of the tube [2,48]. For removing the entrapped nanoparticles, graphene materials can be the premier candidate compared to other carbon nanomaterials like CNTs because of their lower amounts of metallic impurities, as well as the need for less time-consuming purification procedures [52,53]. GO has been known as a two-dimensional material, which is constructed in a crystalline, hexagonal, and single-layer structure by oxygen groups over its surface. This material has a lower thermal and electrical conductivity compared with graphene due to the presence of oxygen groups and the degradation of the main graphene structure. However, these oxygen groups can provide a better capability of interacting with materials [53,54,55,56]. GO has unique intrinsic physical as well as chemical properties and interactions with biopolymers [54,55,56]. Some of the chemical properties include large surface area, functionality containing oxygen, better conductivity and good biocompatibility. The chemical property allows it to be used in bioimaging, biosensing, and hypothermia capabilities [57]. Furthermore, it has appropriate biocompatibility and is utilized in medicine because of its carbonyl, carboxyl, and hydroxyl groups [53,54,55,56]. Figure 4 demonstrates the schematic figure of the morphological structure of CNTs and the single-layer graphene sheet [58].

**Figure 5:**
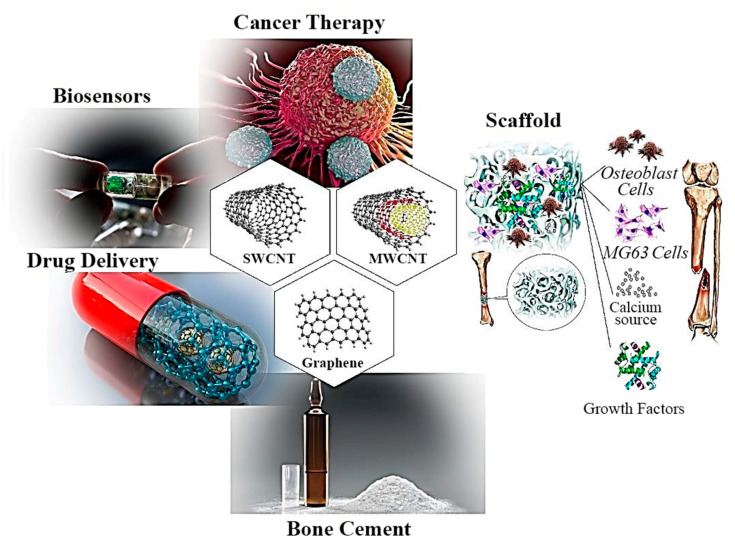
Biomedical application of carbon-based nanomaterial [53,69].

Moreover, GO is utilized in osteogenic stem cells to study [59,60,61] chondrogenesis, adipogenesis, epithelial genesis, myogenesis, cardiomyogenesis, and neurogenesis [62,63,64,65,66,67]. Investigations in the area of CBNs for biomedical applications—like tissue engineering [53], biosensors, drug delivery, cancer therapy [68], and orthopedic BCs [2]—have attracted widespread attention because of their incomparable physical, mechanical, and electrical properties, such as unique shape, large surface specific area, size, thermal, electrical, structural, and optical diversity [68,69,70], which are widely investigated and make them (CBNs) appropriate as reinforcement agents as shown in Figure 5 [53,69]. Many investigations have focused on removing the defects of BCs due to the high utilization of them [71,72,73]. For enhancing and repairing the PMMA-based BCs, many metal, polymer, and ceramic reinforcements such as glass fiber, carbon fiber, kevlar (aramid) fibers, polyethylene fibers, titanium fibers, and metal wires have been utilized [71,72,73]. Among other reinforcements, CBNs like CNTs and GO have been the target of much attention because of their unique mechanical, physical, and chemical properties [71,72,73]. In this paper, we have focused on the incorporation of CNTs and GO in the PMMA-based BCs.

## 4. CNTs in PMMA-Based BCs

### 4.1. Mechanical and Setting Properties of CNTs in PMMA-Based BCs

The first reference found related to the use of CNTs in acrylic BCs is a U.S. patent registered by Pienkowski et al. [74] in 2003 titled “Polymethylmethacrylate augmented with carbon nanotubes”. In this patent, the addition of CNTs to PMMA for BCs and dental composite applications to improve their mechanical performance was proposed. However, the first publication related to the use of CNTs in PMMA-based BCs in a scientific journal was not published before Marrs et al. research in 2006 [75]. In this study, MWCNTs were produced over a substrate of polished quartz in an argon–hydrogen atmosphere [45,76]. The procedure started with injecting xylene in a furnace, considering a controlled rate in the presence of a ferrocene catalyst [76]. This process of chemical vapor deposition needs accurate control of reactor zone temperature, argon–hydrogen atmosphere, catalyst particle size, feed material purity, preheater temperature, catalyst-to-carbon ratio, and so on [75]. These factors (particularly the furnace temperature) can control the diameter, type (single-wall or multi-wall), and length of the obtained nanotubes and also the percentage of unfavorable amorphous carbon. The as-produced MWCNTs were dispersed all over the molten matrix of pre-polymerized commercial BC powder. In this work, different amounts (0.5–10 wt %) of MWCNTs were added to the PMMA-based BCs [75,76,77]. In [77], researchers showed that incorporating MWCNTs into the PMMA-based BCs can significantly enhance the mechanical and fatigue characteristics by an optimum addition of 2 wt % MWCNTs. In a later study, Marrs et al. [78] confirmed that the improvement in the fatigue properties remained after aging the cement in a physiological medium as well as when the solid phase of the cement was modified by the addition of different copolymers. In another study by Nien and Huang [79], mechanical analysis of acrylic BC reinforced by CNTs was evaluated. In their research, MWCNTs with a diameter of 40–60 nm and a length of 0.5–40 mm were used as-received without further treatment and several systems of BC reinforced by CNTs were constructed [78,79]. Firstly, PMMA/CNTs composites were fabricated for achieving better dispersion of CNTs in BCs and then these composites were considered as a powder in BCs, and the specimens of BCs were prepared with blending at the liquid to powder ratio of 1/2 [78,79]. Then mechanical properties of BCs were evaluated by investigating the compressive and tensile properties as well as dynamic mechanical analysis (DMA), and this kind of enhanced BCs showed appropriate material properties like compressive and tensile strength. Their results illustrated the potential of the produced material for utilization in clinical applications. Almost at the same time, Ormsby et al. [80] incorporated MWCNTs into the PMMA-based BCs and evaluated the effects of MWCNTs on the thermal and mechanical properties. In their research, different production methods were compared, and the optimal method that provided more significant improvements in the mechanical properties was selected. PMMA/MWCNTs nano-composite BCs with the incorporation of 0.1 wt % MWCNTs were provided by utilizing three different approaches. The three approaches which suggested embedding the MWCNTs into the cement were as follows: (1) mixing the MWCNTs with PMMA by dry blending, (2) dispersing MWCNTs into the liquid phase (MMA) by magnetic stirring, and (3) ultrasonication using an ultrasonic disintegrator. Mechanical characteristics of the fabricated nanocomposite BCs were affected by the type of MWCNTs along with the utilized incorporation method [80]. The exothermic polymerization reaction in the case of the PMMA-based BCs was dramatically decreased when thermally conductive functionalized MWCNTs were used. The obtained results showed that the best way to prepare the cement with MWCNTs is the dispersion of them into the monomer by ultrasonication just before the preparation of the cement and then the mixing of both cement phases following the conventional procedure [80]. In another study, Ormsby et al. [81] evaluated the effect of MWCNTs loading and functionality on mechanical properties of PMMA/MWCNT-based BCs. In their study, PMMA/ MWCNTs nanocomposite BCs with weight loadings in the range of 0.1–1.0 wt % were provided, and the unfunctionalized, amine, and carboxyl functionalized MWCNTs (MWCNTs-COOH) were analyzed. According to the obtained results, it can be said that incorporating low loadings of MWCNTs (≤ 0.25 wt %) into the PMMA-based BCs can enhance the fabricated nanocomposite mechanical properties. Less enhancements in the mechanical properties are obtained for the higher loadings (≥ 0.5 wt %) [81]. The MWCNTs distribution in the BCs matrix was improved with using chemical functional groups by the MWCNTs-COOH presenting considerable enhancements in mechanical integrity. Also, incorporating MWCNTs-COOH into the liquid monomer by utilizing magnetic stirring was known appropriate in decreasing the values of thermal necrosis index (TNI) under the temperatures between 44 °C and 55 °C to levels below one [81]. It is proposed that the MWCNTs can work as a heat sink in the PMMA-based BCs. Hence, it can assist in the dissipation of the heat generated during the polymerization reaction through enhancing the BC thermal conductivity. In this regard, Ormsby et al. [82] investigated the fatigue and biocompatibility attributes of a PMMA-based BCs reinforced by MWCNTs. The fatigue attributes of these PMMA/ MWCNTs-based BCs were analyzed at the MWCNTs addition amounts of 0.1 and 0.25 wt % by the type and wt % incorporation of MWCNTs utilized possessing the most considerable effect on the cycle number to defect. Agglomerates of MWCNTs were obvious in the cement microstructure and the agglomeration degree was considered to be based on the level of functionality/loading. Ormbsy et al. [81,82] investigated the effect of MWCNTs with different functionalities (unfunctionalized, carboxyl functionalized, and amine functionalized) on mechanical properties. In another study by Ormbsy et al. [83], the effect of MWCNTs incorporation on rheological and thermal properties of PMMA-based BCs was analyzed. In this work, composites of MWCNTs/PMMA by loadings in the range of 0.1–1.0 wt % were provided as BCs. Unfunctionalized, carboxyl, and amine-functionalized MWCNTs were also utilized. The results demonstrated that MWCNTs incorporation dramatically changed the reaction of polymerization and cure kinetics of the achieved PMMA-based BCs [83]. The extent of this influence was related to the chemical functionality, dispersion, and MWCNTs loading into the cement microstructure [83]. The polymer gelation and onset of polymerization can change as a function of loading and the MWCNTs type. The MWCNTs network in the PMMA microstructure proved a physical and chemical interaction during the polymerization reaction [83]. An adequate functionalization of the surface of the nanoparticles promotes this interaction between the nanoparticles and the BC matrix and these interactions can help the loss of heat energy produced during polymerization besides prolonging the free radical reaction [83]. Therefore, chemical functionalized MWCNTs presented the most considerable reduction in the extent of the exothermic reaction. They show better chemical interaction by the polymerization reaction of PMMA-based BCs in comparison with their unfunctionalized MWCNTs counterpart. In a different work, Ormbsy et al. [84] analyzed carboxyl functionalized MWCNTs/PMMA-based BCs for orthopedic usage. They investigated the incorporation of 0.1 wt % MWCNTs-COOH into the Simplex P ^TM^ (Stryker Howmedica Osteonics, Republic of Ireland) BC and showed that mechanical properties of the Simplex P^TM^ BC were enhanced by adding 0.1 wt % of MWCNTs-COOH [84]. The uniform dispersion of MWCNTs-COOH into the Simplex P^TM^ BC delayed crack propagation through the cement mantle during fatigue and static loading [84]. The higher incorporation of MWCNTs-COOH can dramatically decrease an exothermic polymerization reaction, which is due to the fact that the MWCNTs-COOH is working as a heat sink in the BC matrix. According to the results of Raman spectroscopy, it can be said that a chemical interaction between the Simplex P^TM^ BC and MWCNTs-COOH happened [84]. The mentioned interaction facilitates the chemical bonding of the MWCNTs-COOH to Simplex P ^TM^, hence allowing the favored transfer of the mechanical load. The results of these studies show that the polymerization reaction of the cement is affected by the use of the MWCNTs; in this regard, the maximum temperature reached during the curing process decreased, and the reaction was delayed, resulting in the increase of the setting time. These effects were more significant as it increases the load of MWCNTs [16]. Xu et al. [85] presented an effective method for the functionalization of MWCNTs based on nitric acid oxidation. The surface modification of MWCNTs was successfully performed by employing an HNO_3_ hydrothermal functionalization approach. The results proved that adding 0.6 wt % functionalized MWCNTs to the PMMA-based BCs can improve both the bending and compressive strengths of the obtained nanocomposite [85]. According to the obtained results, it can be stated that enhancements in mechanical properties are because of the MWCNTs arresting/retarding propagation of crack via the BC using a bridging influence as well as hindering crack propagation and appropriately homogenous distribution of MWCNTs in the PMMA-based BCs, which can attract and retard crack propagation in the cement [80,81]. Achieving a good dispersion is essential for an adequate interaction between the MWCNTs and the matrix, favoring the load transfer between the MWNCTs and the matrix and delaying the crack propagation through the cement mantle [81,82]. On the other hand, reductions that can be seen in mechanical properties are due to MWCNTs agglomerations happening in the microstructure of the cement, and the degree of these agglomerations is related to the approach utilized for incorporating the MWCNTs into the cement [80,81]. At this point, achievement of an adequate dispersion of the nanoparticles in the polymeric matrix of the cement is one of the key points to obtain an enhanced MWCNTs/PMMA-based BCs. Besides, for many cases, considerable reductions were seen by the higher percentage of MWCNTs incorporation [80,81,82,83,84,85]. In [86], researchers investigated a conducted crack in CNTs reinforced BC using a finite element model to perform the fracture analysis of PMMA/CNTs composite material. This study considered the production of CNTs interacting by a pre-existing crack instead of the traditional model of a single fiber. The influences of geometric factors of the CNTs, as well as the material structural heterogeneity over crack propagation trajectory were evaluated [86]. It was determined that the fracture behavior was considerably affected by material properties and the morphological parameters of CNTs. The obtained results proved that at the crack tips, CNTs could affect the crack and cause a reduction in the stress intensity factor (SIF). Nevertheless, it can be limited to the proximity of the CNTs [86]. The trajectory of crack growth is additionally affected by the nano-structural adjustment of the composite components. Above all, the results clearly showed that the CNTs could either decline or absorb the crack depending not on their dispersion along with adjustment but additionally on the change of their material properties [86]. It was found that the CNTs act as an obstacle to the crack growth, which can be a great advantage for clinical applications. In another study, Lin et al. [87] analyzed the influences of MWCNTs over fatigue attributes of PMMA-based BCs in which they investigated the fatigue properties of MWCNTs-PMMA-based BCs composites. In this research, the mixing quantity of MWCNTs in the range of 0.1–1 wt % was incorporated into the PMMA-based BCs. The researchers proved that regardless of the MWCNTs type or the chemical functionality utilized, even a low MWCNTs loading of 0.1 wt % can yield excellent enhancements [87]. Moreover, MWCNTs-COOH can considerably enhance the fatigue life of the composite cement. Pull-out and bridging were recognized as the two reasons for observed reinforcement in the MWCNTs/PMMA-based BCs. The enhancements of fatigue properties were due to the considerably uniform dispersion of MWCNTs in the BCs, which can retard or arrest crack propagation in the BCs by bridging cracks and relieving stress through the pull-out of MWCNTs [87]. The distribution of MWCNTs was improved with modification by chemical functional groups, preparing the most considerable enhancements in mechanical properties. It was assumed that improved distribution can promote an enhancement in chemical interaction between the cements and MWCNTs. Arun et al. [88] studied mechanical properties of PMMA/SWCNTs-based BCs by utilizing nano-indenter. In the stem/cement interface, the huge difference between the mechanical properties of the cortical bone and BC caused inhomogeneous stress transfer. Therefore, an attempt was made for sorting out the above-mentioned issue by reinforcing PMMA-based BCs by SWCNTs. Firstly, the SWCNTs were functionalized by alkaline treatment [88]. The attachment of sodium ethoxide was proved by using Fourier transform infrared spectroscopy (FTIR). Then, polymerization was performed with the reinforcement of SWCNTs and mechanical properties were evaluated utilizing the nano-indenter. Their results illustrated that the Young’s modulus and hardness of PMMA were improved by 19% and 36%, respectively, at an optimized 0.15 wt % SWCNTs concentration [88]. The improvement of mechanical properties was because of the discrete dispersion of SWCNTs in the PMMA matrix as well as the impressive load transfer from the matrix to the reinforcement. Besides the mentioned investigations, many works have been done in the field of the influences of CNTs composite on PMMA-based BCs. Besides, Pahlevanzadeh et al. [89] developed PMMA-monticellite (Mon)-CNTs-based BCs with great mechanical characteristics and desirable biological attributes for utilizing in bone-defect treatment. They synthesized and characterized a new PMMA-based BCs comprising CNTs and Mon for the treatment of bone defect and proved that more appropriate mechanical properties were obtained in the PMMA-Mon-CNTs composite compared to the PMMA and PMMA-Mon-based BCs because of the unparalleled resistance of CNTs to crack formation and propagation [89]. In another study, Soleymani et al. [90] studied the influences of chitosan (CS)/MWCNTs composite on mechanical, biological, and physical properties of the PMMA-based BCs. They prepared CS/MWCNTs composite powder and CS powder and added them to the PMMA-based BCs in different concentrations considering a uniform dispersion [90]. Their results demonstrated that incorporating CS/MWCNTs into the PMMA-based BCs can enhance the injectability and setting time while the contact angle and maximum temperature were remarkably reduced. Mechanical analysis proved that the compressive and bending strengths were notably enhanced by 25 wt % incorporation of the CS/MWCNTs composite powder into the PMMA-based BCs [90].

### 4.2. Biological Properties of CNTs in PMMA-Based BCs

Wang et al. [91] analyzed the incorporation of MWCNTs into the PMMA-based BCs and its influence on osseointegration and cytocompatibility. The in vitro study proved that the DNA content, osteocalcin gene expression, osteopontin, protein/DNA, and ALP/DNA of the rat bone marrow mesenchymal stem cells (rBMSCs) over the MWCNTs-incorporated PMMA-based BCs were dramatically enhanced in comparison to the cement without MWCNTs [91]. The extent of enhanced content was straightly proportional to the MWCNTs loading level. It was proposed that the incorporation of MWCNTs not only improved cell adhesion but also induced osteogenic differentiation [91]. Another study suggested by Ormbsy et al. [82] showed that the biocompatibility of the MWCNTs/PMMA-based BCs at MWCNTs loading amounts lower than 1.0 wt % was specified by considering the appointed biological cell culture study utilizing MG-63 cells [82]. In this regard, the results demonstrated that MG-63 osteoblastic cells appropriately acted and proliferated on the surfaces of all MWCNTs/PMMA-based BCs compositions analyzed during the 7-day time period. It was determined that no variations were seen in the morphology of the cells cultured over the MWCNTs/PMMA-based BCs in comparison with those on the control cement. In another study, Goncalves et al. [92] studied the in vitro biocompatibility of PMMA/high-load HA/CNTs-based BCs formulations. In this work, by considering in vitro biocompatibility, two optimal compositions with GO (0.5% w/w) or a small amount of functionalized (f)-CNTs (0.1% w/w) and a high concentration of HA (67% w/w) in their formulation have been evaluated as mechanical reinforcement phase [89]. The results indicated the ability of these novel materials to promote the growth of the Ca-P layer on the surface of the BCs discs, increase cell viability, decrease apoptosis, and extend the spread on the disc surfaces. Hence, the mentioned BCs formulations can have the potential to be used in clinical applications. According to the obtained results of Pahlevanzadeh et al. [89], Desirable bioactivity was determined in the Mon and CNTs-based BCs, while the PMMA-based BCs showed low bioactivity. The incorporation of CNTs and Mon within the PMMA matrix enhanced the attachment of the MG-63 cells in comparison to the PMMA-based BCs. Therefore, in orthopedic surgeries, the PMMA-Mon-CNTs-based BCs have shown a good potential as a candidate for filling the bone defects. In this regard the results of Soleymani et al. [90], showed that the incorporation of 25 wt % CS/MWCNTs composite powder into the PMMA-based BCs can remarkably increase the apatite like deposition and Extra-cellular matrix (ECM) formation, based on the cellular activity and bioactivity analysis. Hence, 25 wt % PMMA-CS/MWCNTs composite offered a novel approach for enhancing commercial PMMA- based BCs.

## 5. Graphene (G) and GO in PMMA-Based BCs

### 5.1. Mechanical and Setting Properties of Graphene (G) and GO in PMMA-Based BCs

In the presence of GO in polymers, the stress transfer from the polymer matrix to GO can occur, and elastic modulus, strength, and toughness can be increased because of chemical bonding with the polymer [93,94]. Paz et al. [95] analyzed graphene and GO to optimize PMMA-based BCs for orthopedic usages. They added nano-sized GO and graphene powders in the range of 0.1–1.0 wt % as reinforcement to PMMA-based BCs in which BCs have provided to contain powders of GO and graphene. Firstly, the GO or graphene powders were distributed in the liquid phase by utilizing an ultrasonic disintegration. After that, the liquid and powder phases were mixed based on the instructions at ambient temperature as well as at a relative humidity of not less than 40% by using a commercial vacuum mixing device [95]. Mechanical properties, particularly the fracture fatigue and toughness characteristics, of the GO/PMMA and G/PMMA-based BCs were enhanced at low concentrations of ≤0.25 wt %. The mentioned enhancements were because of the effects of GO and graphene on deviations of crack tips and formation of an obstacle in crack propagation [95]. The high functionalization of GO in comparison with graphene yielded greater improvements since it allowed to make a stronger interfacial adhesion between the PMMA and GO. Using ≥0.25 wt % concentrations caused a decrease in mechanical characteristics due to the formation of agglomerates and increase of porosity. Due to the thermal properties, the level of unreacted residual monomer raised, and the produced polymerization heat was reduced when the GO and graphene loading raised from 0.1 to 1.0 wt % [95]. These mentioned influences were due to the active role that the carbon-based nanomaterial such as GO and MWCNTs played during the polymerization reaction as a ‘radical scavenger’ because of the double bonds which are converted to reactive forms and affected the free radicals during the polymerization of BCs. In a study by Paz et al. [96], GO and graphene with silanes were used for advanced distribution and strengthening of PMMA-based BCs. They considered an appropriate way for the functionalization of graphene, through silanization by [3-(Methacryloyloxy) propyl] trimethoxysilane (MPS) which improved dispersibility of the graphene sheets, preparing a considerable improvement in mechanical and dispersibility properties. Their results exhibited that the silanization considerably enhances the graphene dispersibility: after 24 h, the pristine graphene dispersion fell, and after five days, the silanized graphene illustrated adequate stability. In addition, it could improve dispersibility and generate a notable enhancement in mechanical characteristics of the graphene-reinforced BCs [96]. The bending and compressive strengths of silanized graphene enhanced by around 13.7% and 12%, respectively, and the toughness of fracture around 28% compared with the pristine graphene. The enhancement in the efficacy of reinforcement of the graphene post-silanization was ascribed to two reasons: (1) in the MMA suspension, silanization enhances the long-term dispersion of the graphene and can hence decrease the trend of aggregation formation, (2) the approach of silanization improves the formation of the covalent bond at the interface of graphene-matrix and, as a result, produces a firmer adhesion between the matrix and graphene following polymerization [96]. Despite the remarkable results obtained concerning graphene, the route traced in the case of the GO silanization was not beneficial which was due to the formation of agglomerates as well as a loss of nanoparticle porosity in post-silanization [96]. Mechanical, physical, thermal, and chemical attributes of nano-scale GO-PMMA composites were studied by khan et al. [97]. This work aimed to analyze and model the GO-PMMA resin composite by the considered BC. They fabricated GO via the approach of ultrasonication, and three different groups were analyzed in which 0.024% (w/w) of GO was incorporated into a matrix of resin for GO1-group, 0.048% (w/w) of GO was added to the matrix of resin for GO2-group and the control group as C-group was provided. Mechanical and physical properties of the samples were analyzed and the obtained results demonstrated that 0.048% (w/w) and 0.024% (w/w) of loaded GO has no influence on the physicochemical properties, but thermal properties could be enhanced, slightly [97]. The bending strength and wear resistance of GO1 and GO2 groups considerably enhanced in comparison with the C-group. It was stated that the use of GO-PMMA composites could positively increase the mechanical properties of BC. In a study, Ahmed Khan et al. [98] evaluated the dynamic and static mechanical characteristics of the GO-based BCs agents. Their aim was to formulate the GO nano-sheets as well as to characterize the composites containing homogeneously dispersed sheets of GO in the PMMA acrylic resin of two groups (by 0.025% w/w of GO1 and 0.05% w/w of GO2 groups). A large array of surface, mechanical, and dynamic mechanical characteristics were evaluated. Their results showed that as a BC alternative, GO loading promoted the mechanical properties of PMMA, enhancing surface hardness at the cost of enhanced hydrophilicity [98]. A higher amount of GO reinforcement caused higher porosities and enhancement of the stiffness on the fabricated samples. The addition of GO in PMMA can make a material that is robust, mechanically, and stable, thermally [98]. In order to enhance the mechanical and physical properties of the PMMA/GO composite BCs, many researchers have added composites of GO to the PMMA-based BCs. In a research, Gonçalves et al. [99] analyzed the influence of GO on bioactive PMMA/ HA-based BCs. They added GO to a composite matrix of PMMA/HA-based BCs, considering the loading in the range of 0.01–1.0 wt %. The preparation approach included the use of nano-fillers on the solid fraction of the BC due to homogenization in aqueous suspension that was frozen granulated for maintaining the identical dispersion of all the ingredients and dried using a lyophilization process [99]. Then the nanocomposite cements were provided by utilizing the conventional method of mixing liquid and solid phases, promoting the PMMA in situ radical polymerization, keeping the solid to liquid loadings relation of the conventional BC unchanged. It was found that for the PMMA radical polymerization, GO possesses an active interposition by using as a radical scavenger during the PMMA polymerization reaction because of the delocalized π-bonds [99]. As a result, there exist retardation and prevention of the polymerization that influences the final mechanical characteristics of the nanocomposite. For suppressing this drawback, radical concentration applied during the PMMA bulk polymerization was raised for overcoming the percentage of radicals inactivated with GO [99]. It was determined that mechanical properties of the resultant nano-composites demonstrate a considerable enhancement with doubling the initial radicalar agent concentration. In addition, the most significant result was obtained for a GO reinforcement of 0.5 wt %. [99]. In this regard, Pahlevanzadeh et al. [100] reported that in the bone cement including PMMA–polycaprolactone (PCL)–fluorapatite (FA)– GO which was produced as bone filler, both the compressive strength and elongation enhanced dramatically after the use of GO within the PMMA-PCL-FA-based BCs. In a study by Tavakoli et al. [101], mechanical, biological, and physical properties of the PMMA-CS/GO composite BCs were investigated. The researchers, after providing CS and CS/ GO powders, added them homogeneously to the PMMA-based BCs in different concentrations (20, 25, and 30 wt %). The results demonstrated that incorporating 25 wt % of CS/GO powder into the PMMA-based BCs leads to the enhancement of the compressive modulus around 69.1% and the compressive strength by about 16.2% as well as the bending strength by 24.0%. Based on the physical evaluations [101], using the CS/GO powder on the PMMA-based BCs, the adjusting time and injectability were enhanced and the highest temperature was reduced. In another study, Valencia Zapata et al. [102] suggested a new antibacterial and bioactive acrylic bone cements (ABCs) nano-composite enhanced by GO and CS. They showed that the thermal and mechanical properties of ABCs improved by CS and GO. The results demonstrated the appropriate dispersion of GO nano-sheets in the ABCs [102]. The nano-sheets provided an enhancement in roughness and flexural behavior, whereas CS produced porosity, enhanced the degradation rate, and reduced compression properties [102]. The new formulation of ABCs, including CS and GO, simultaneously enhances the flexural modulus and thermal stability which provide it with a high possibility to be utilized in orthopedic applications. In a different study, Goncalves et al. [103] investigated GO vs. f-CNTs as reinforcements in a PMMA/HA-based BCs. They studied f-CNTs and GO (each in the concentration in the range of 0.01–1.00% w/w) as the reinforcing agent in a PMMA/HA-based BCs. Concerning the GO-reinforced cements, higher values in mechanical properties were found in comparison with the corresponding amounts for the unreinforced cement [103]. It can be due to the high degree of surface functionalization GO, high specific area, and wrinkled surface that can cause high adhesion/interlocking for the nano-filler by the BCs matrix [103]. There exists depreciation whether or not the two nano-fillers are utilized for other mechanical properties specified, and it can be said that the carbon nanostructure operates as a scavenger of the radicals which are generated during the process of polymerization, leading to retardation and inhibition of the formation of polymer chains [103]. It is more observed when f-CNTs were utilized compared to when GO was utilized due to the higher density of the p-bond area. According to this statement, it was suggested that one method, which can be used for countering the negative influence of carbon nanostructure on mechanical characteristics of the BCs, was to enhance the radical initiator concentrations (RICs), substantially, in the cement during polymerization [103]. In another investigation, Sharma et al. [104] compared osteoconductive ability of G/PMMA-based BCs composites with controlled PMMA-based BCs. Furthermore, they have evaluated setting characteristic of BCs encapsulated with graphene, GO, and surface treated amino graphene (AG) as a reinforcement agent. Their finding revealed that AG-based nanohybrids considerably diminished the exothermic curing temperature to body system and amplified the setting time to assist in practitioner, indicating that setting characteristic could be effectively manipulated through different the amount of the reinforcement agent. In another study Paz et al. [105] analyzed biocompatibility, antimicrobial activity, and thermal properties of GO and graphene reinforced PMMA-based BCs. They incorporated 0.1 wt % of graphene or GO powders into the PMMA-based BCs, which was introduced as an optimal loading level in terms of enhancing the mechanical performance in the previous studies [105]. Their results demonstrated that at a loading level of 0.1 wt %, the association of graphene or GO powder to PMMA-based BCs was suitable for utilization during joint replacement surgery. They proved that the thermal properties of PMMA-based BCs did not show any considerable variation with the addition of graphene or GO powders [105]. Table 1 and Table 2 exhibit the mechanical and setting characteristic of the PMMA-based composite BCs.

### 5.2. Biological Properties of Graphene (G) and GO in PMMA-Based BCs

**Figure 6:**
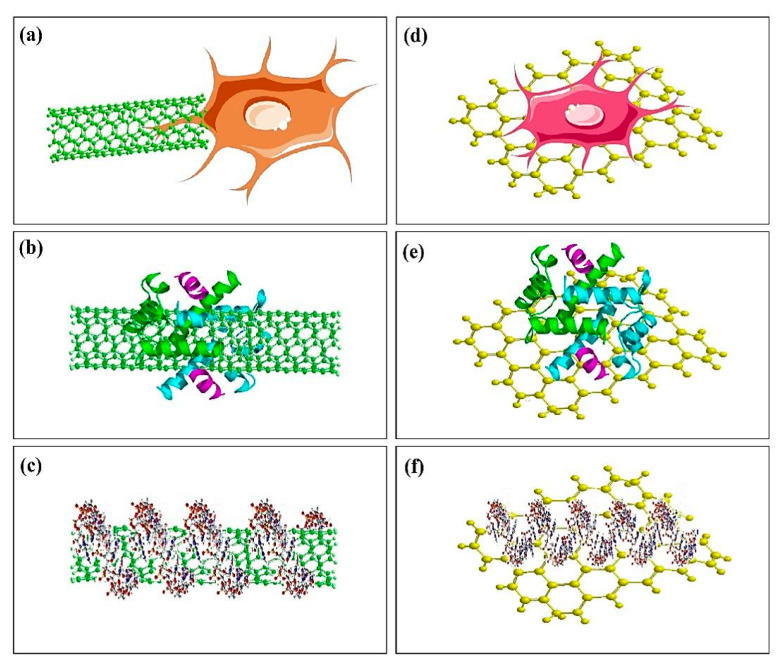
Schematic figure of interactions of CNTs and graphene with biological systems: (**a**) interaction of CNTs with cell, (**b**) adsorption of protein biomolecules on CNTs, (**c**) interaction of nucleic acids with CNTs, (**d**) interaction of graphene with cell, (**e**) adsorption of protein on graphene, and (**f**) interaction of nucleic acid with graphene [106].

In the study that Paz et al. [105] analyzed biocompatibility, antimicrobial activity, and thermal properties of GO and graphene reinforced PMMA-based BCs, the obtained results showed that the GO/PMMA and graphene/PMMA-based BCs presented no cytotoxic response and sufficient level of biocompatibility. Nevertheless, at a constant level of loading equal to 0.1 wt %, the incorporation of graphene or GO powder did not show an enhancement in antimicrobial activity of the PMMA-based BCs [105]. According to the results of Gonçalves et al. [99], the biocompatibility of this material was analyzed in vitro by using mouse L929 fibroblasts as well as human Saos-2 osteoblasts cultured for 3 days in contact with rough and smooth surfaces of disks provided by the novel composite. Both cell kinds adhere and then grow on all these surfaces by high cell viability [99]. It was concluded that PMMA-based BCs enhanced by GO were potential candidates to be utilized as BCs by promoted mechanical properties along with allocated biological conduct The obtained results in case of bioactivity test for the composite proved the importance of HA presence in this composite for promoting the bioactive behavior. In addition to the discussed studies, Pahlevanzadeh et al. [100] investigated a bone cement including PMMA-PCL-FA-GO which was produced as bone filler for application in orthopedic surgeries and evaluated in-vitro biocompatibility, bioactivity, and mechanical properties of this composite BCs. The researchers homogeneously distributed the GO and FA particulates in the PMMA-PCL polymer matrix and revealed that the incorporation of GO and FA into the polymer cement (i.e., PMMA-PCL) improved the apatite formation capability on the polymer surface. The results showed that the viability of MG-63 osteoblast cells enhanced after the use of GO and FA in the PMMA-PCL polymer BCs [100]. In this regard, Tavakoli et al. [101] reported that by adding 25 wt % CS/GO composite powder to PMMA-based BCs, apatite-like deposition was enhanced [101] and the results in the case of MG-63 cell culture proved the improvement of growth, cell adhesion, and cell viability on 25 wt % for PMMA-CS/GO composite BCs. In the study concluded by Valencia Zapata et al. [102], it was shown that biological and physicochemical properties of ABCs improved by CS and GO and nano-sheets provided an enhancement in antibacterial activity. All of the considered ABCs are non-cytotoxic and can properly support cell viability of the human osteoblasts (HOb) and simultaneously enhances osteogenic activity, and antibacterial behavior [104]. In this regard, Sharma et al. [104] reported that AG based nanohybrids have shown greater osteointegration and lower cytotoxicity as compared to other nanohybrids as well as pristine BCs. Thus, this newly developed nano-composite can create natural bonding with bone tissues for improved bioactivity, longer sustainability, and better strength to the prosthesis. Definitely biomedical use of carbon-based materials, including CNTs and GO, mainly rely on knowing and essential evaluation of the mechanisms of their interactions with numerous biomolecules in the organic environment, including DNA, RNA, and proteins [106]. These analyses utilized different analysis methods to significantly evaluate the interaction of CNTs and graphene in the organic environment. For instance, atomic force microscopy (AFM) was carefully employed to examine the biofunctionalization of CNTs and graphene, and their interaction with different organic membranes, including nuclear and cell walls [107,108]. Use of AFM is particularly helpful in the recognition of living cells, cellular response, and their physiological characteristics. As a consequence of their small size, graphene and CNTs have the possibilities to get into several cellular areas such as the nucleus [106]. A current study of this finding via Porter et al. [109] further outlined the alteration of CNTs throughout the nuclear membrane layer and their existence in the nucleus. This stimulates the use of CNTs as appealing additive phase for delivery of bioactive molecules including DNA and RNA regarding their healing rate particularly within the nucleus of the cell. Figure 6 [106] shows a schematic illustration of interaction of various biological systems such as, cells, nucleic acids, and proteins with CNTs and graphene [106]. Thus it is particular interest to observe the binding of graphene and CNTs with various biological species. Table 3 shows cellular responses of the PMMA-based BCs.

**Figure 7:**
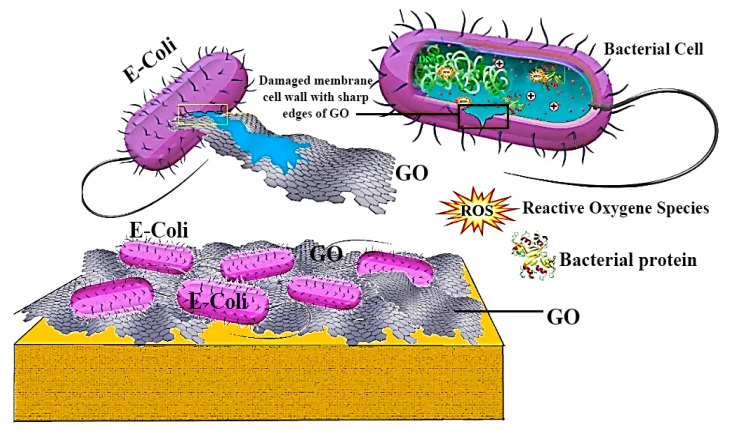
Schematic demonstration of the antibacterial activity of GO-based materials and killing activity of the GO towards bacteria [112].

The antibacterial performance of graphene was initially reported in 2010 [110], which graphene was observed to significantly destroy and inactivate the bacteria attachment with it [110]. This antibacterial process was productive and fast to *E. coli* and *S. aureus*, and the destroy was observed to primarily happen in the cell membrane. A test pointed out that graphene nanosheets actually possessed a greater antibacterial performance in comparison with conventional antibiotics, including kanamycin [110,111]. Similar to graphene, GO likewise exhibits an outstanding bactericidal performance to an extensive range of bacteria [110,111]. On the whole, GO can destroy human bacterial pathogen as a result of its plentiful functional groups together with its fine size; subsequently, GO might cause membrane dysfunction and destruction right after direct attachment; lastly, GO results in killing the cell as can be seen in Figure 7 [112]. Nevertheless, cellular damage could possibly take place through the effect of physical membrane dysfunction which is actually recognized as the biological influence of CNTs. This illustrates that bacterial cells might be irreversibly destructed after a direct attachment to CNTs-based materials [112]. The characteristics and antibacterial behavior of CNTs and GO-based materials are displayed in Table 4 and described in the subsequent sections.

## 6. Strengthening Mechanisms and Future Outlook of the PMMA-Carbon-Based BCs

**Figure 8:**
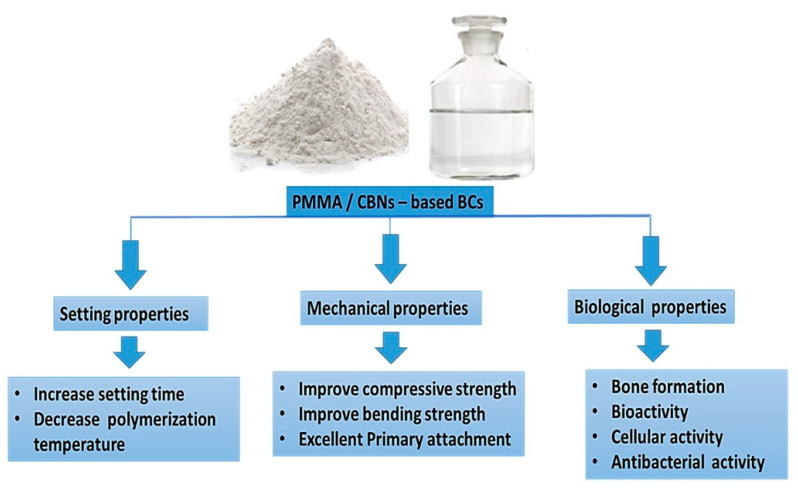
The future trend of PMMA/CBNs-based BCs.

It is an important finding that the mechanical failure of the BCs mantle stays a big issue in the case of joint replacement surgery [9,124]. Mechanical failure of PMMA-based BCs was considered to be accomplished in three stages as is the case with typical fiber-reinforced composites [9]; (a) crack initiation because of an initial defect in the material stability, (b) slow growth of the crack, and/or (c) fast propagation to fracture [9,124]. The use of MWCNTs to the liquid monomer by utilizing magnetic stirring is demonstrated to have a negative influence on the mechanical performance of BCs. It can be due to the negligible distribution of MWCNTs in the liquid monomer that can result in MWCNTs agglomerations in the matrix of the cement [9,124]. In the cement, the above mentioned agglomerations can act as stress concentrations, presenting a mechanism for precocious failure of the cement while related to a loading regime. In the obtained nanocomposite, the disintegrating of the MWCNTs and the dry mixing of the MWCNTs in the polymer powder in the liquid monomer by utilizing ultrasonic agitation enhanced the disentanglement of the nanotubes and facilitated more homogeneous distribution of the MWCNTs [9]. In one study, Andrews and Weisenberger [125] presented that ultrasonic distribution was an impressive technique for the MWCNTs distribution at low levels (<5 wt %) of loadings [126]. Nevertheless, Marrs et al. [77] showed that care is required for distributing MWCNTs in a polymer matrix. In addition, they provided the details of the adverse influences of incompletely scattered clumps of MWCNTs, especially for the reinforcement concentrations higher than 5 wt %. Based on the previous studies, it has been determined that considering well distributed MWCNTs in the PMMA-based BCs by their foretasted strong nanotube matrix bonding as well as high tensile properties, a percentage of the MWCNTs can be orientated by their longitudinal axis vertical to the crack wave [77]. MWCNTs are impressive in bridging the initial crack and barricading crack propagation, further increasing the longevity of the cement mantle with enhancement in mechanical properties. The interface of filler/matrix in the fiber-reinforced polymer composites is important in adjusting the load transfer from the matrix to the fiber and mechanisms of failure as well as degradation [80]. In a study, Gojny et al. [126] stated that considering chemical functional groups to the MWCNTs can enhance a negative charge for the MWCNTs. Therefore, it can decrease agglomeration and enhance the interaction between the host polymer and the nanotubes. The PMMA-based BCs, along with unfunctionalized MWCNTs, showed the lowest remarkable enhancements (*p*-value\0.05) in all measured mechanical properties [126]. It can reduce the mechanical properties due to the poor distribution of MWCNTs in the matrix of cement, causing agglomerations of MWCNTs. Besides, based on the obtained results related to PMMA-based BCs reinforced by GO, the statistically more considerable compressive strengths of the PMMA/GO composite BCs in comparison with PMMA-based BCs considering wet and dry storage conditions may be attributed to two potential mechanisms [126]. In the first step, the presence of enough functional groups, like carboxylic and hydroxyl groups, on the GO surface can help the interfacial interactions between PMMA and GO, which simplified the creation of stronger interfacial adhesion between PMMA and GO resin [98,127]. It should be noted that the wrinkled GO surfaces can improve the eventuality of mechanical interlocking to a greater extent [128,129,130,131,132]. Taken together, future research can focus on the characterization of the PMMA-carbon-based composite BCs system and the effect of CBNs including graphene, GO, and CNTs combinations for future therapeutic advancements and fractured bone treatments development. The encapsulation of CBNs into PMMA-based BCs may enhance various characteristics of the BCs. Therefore, these PMMA/CBNs-based BCs suggested improving the setting properties, mechanical properties, and biological characteristics including acceleration healing rate as shown in Figure 8.

## 7. Conclusions

PMMA-based BCs can be conveniently shaped and adapted to complex bone cavities or used in orthodontic applications to repair dental damages [133,134]. Compared to other comparable materials, the main advantages of utilizing PMMA-based BCs are the excellent primary attachment between the bone and the implant and the patients’ quicker healing. Despite the remarkable success rate of implant fixation with BCs, they have some drawbacks, such as lack of bone formation and bioactivity, low mechanical properties, local tissue damage due to exothermic polymerization reactions, and the mismatch of stiffness between the bone and the cement [135,136,137,138,139,140,141,142,143]. A further issue is the BC failure, which is the main reason for its mechanical malfunction and the aseptic loosening of the implants. It has been demonstrated that PMMA-based BCs cannot form covalent bonds with the natural bone, and a lack of interactions may induce implant loosening after a while. According to the results, the addition of CNTs, GO, and carbon-based composites [144,145,146,147,148,149,150,151,152,153,154,155,156,157,158,159,160,161,162,163,164,165,166] can enhance the setting times, temperature, strength, bioactivity, and cellular activity of the PMMA-based BCs. However, it is believed that utilization of GO could have further impact than CNTs reinforcing agents on enhancement of the strength and durability of PMMA-based BCs, conquering the potential issue presented by premature failure of the implant.

## Figures and Tables

**Table 1:** Mechanical properties of the PMMA-based BCs containing CNTs (MWCNTs and SWCNTs), CS/MWCNTs, GO, PCL-FA-GO and CS/GO reinforcing agents.

Sample	Compressive Strength (MPa)	Compressive Modulus (GPa)	Bending Strength (MPa)	Bending Modulus (GPa)	Fracture Toughness (KIC) (MPa.m1/2)	Ref.
PMMA/MWCNTs (with 2 wt % MWCNTs)	-	-	90.6 ± 3.2	3.52	1.23 ± 0.22	[77]
PMMA-PMMA/CNTs (17/3 g/g)(in PMMA/CNTs ratio was 100/0.2 g/g)	130.16 ± 3.83	-	-	-	-	[79]
PMMA/functionalized MWCNTs (with 0.1 wt % MWCNTs)	62.24 ± 3.60	3.21	68.48 ± 9.39	3.26	1.49 ± 0.12	[80]
PMMA/MWCNTs (0.1 wt % carboxyl functionalized MWCNTs added to MMA pre-polymerization)	86.5 ± 6.6	4.8	68.8 ± 5.6	3.5	1.5	[81]
PMMA/MWCNTs-COOH (with 0.1 wt % MWCNTs)	104.47 ± 6.05	3.67	102.88 ± 19.26	6.12	3.07± 0.39	[84]
PMMA/MWCNTs (with 0.6 wt % MWCNTs) Ultrasonic disintegration	92.9 ± 2.2	-	74.1 ± 2.2	-	-	[85]
PMMA/SWCNTs (with 0.15 wt % SWCNTs)	-	4.58	-	-	-	[88]
PMMA-CS/MWCNTs (with 25 wt % CS/MWCNTs containing 2.5 wt % CS and 0.5 wt % MWCNTs)	127.33 ± 7.41	1.67	107.80 ± 2.30	5.7	-	[90]
PMMA/GO (with 0.1 wt % of GO)	120.7 ± 16.1	-	66.4 ± 6.50	3.29	2.17 ± 0.11	[95]
PMMA/ GO (with 0.1 wt % GO)	81	-	57	-	1.53 ± 0.07	[96]
PMMA/GO (with 0.048 wt % of GO)	-	-	87.0 ± 7.2	-	-	[97]
PMMA/GO (with 0.05 wt % of GO corresponding to MMA monomer)	90.94	4.4	-	-	-	[98]
PMMA-PCL-FA-GO	138 ± 7	0.5	-	-	-	[100]
PMMA-CS/GO (with 25 wt % CS/GO containing 2 and 0.3 wt % CS and GO respectively)	93.0 ± 4.0	1.21	79.9 ± 1.8	-	-	[101]
PMMA/CS/GO (with 15 wt % of CS and 0.3% of GO)	77	-	-	-	-	[102]

**Table 2:** Setting properties of the PMMA- based BCs containing MWCNTs, CS/MWCNTs, GO, CS/GO, and AG reinforcing agents.

Sample	T Max (°C)	Setting Time (sec)	Ref.
PMMA/ functionalized MWCNTs (with 0.1 wt % MWCNTs)	68.53 ± 6	1712 ± 7	[80]
PMMA/MWCNTs (with 1 wt % MWCNTs)	≈57	≈1100	[83]
PMMA/ MWCNTs-COOH (with 0.1 wt % MWCNTs)	70.50 ± 6.87	694.2 ± 16.74	[84]
PMMA-CS/MWCNTs (with 25 wt % CS/MWCNTs containing 2.5 wt % CS and 0.5 wt % MWCNTs)	58.10 ± 2.77	970.2 ± 48	[90]
PMMA/GO (with 0.1 wt % of GO)	79	1080	[95]
PMMA-CS/GO (with 25 wt % CS/GO containing 2 and 0.3 wt % CS and GO respectively)	62.3 ± 1.9	768 ± 20.4	[101]
PMMA/AG	38 ± 1	1320 ± 120	[104]

**Table 3:** Cellular responses of the PMMA-based BCs containing MWCNTs, CS/MWCNTs, HA/GO, HA/CNTs, PCL-FA-GO, CS/GO, AG, G and GO reinforcing agents.

Sample	Cellular Assay	Cell Type	Target Tissue	Application	Ref.
PMMA/MWCNTs	MG-63 osteoblastic cells successfully adhered to and proliferated on the surfaces of all MWCNTs–PMMA cement	Osteoblast-like MG-63 cells	Bone	In vitro	[82]
PMMA-CS/MWCNTs (with 25 wt % CS/MWCNTs containing 2.5 wt % CS and 0.5 wt % MWCNTs)	The activity of the osteocyte cells leads to the formation of the ECM.	Human osteosarcoma cell line MG-63	Bone	In vitro	[90]
PMMA/MWCNTs	Promoted cell adhesion, induced osteogenic differentiation, Promoted osseointegration,	Bone marrow-derived mesenchymal stem cells (rBMSCs)	Bone	In vitro	[91]
PMMA/ HA (67 wt %)/GO (0.5 wt/wt %)	Induce calcium phosphate high cell viability, low apoptosis, and extensive spread on disc surfaces	L929 fibroblasts and human Saos-2 osteoblasts	Bone	In vitro	[92]
PMMA/HA (67 wt %)/functionalized CNTs (0.1 wt/wt %)	Induce calcium phosphate high cell viability, low apoptosis, and extensive spread on disc surfaces	L929 fibroblasts and human Saos-2 osteoblasts	Bone	In vitro	[92]
PMMA- HA/GO	Adhere and then grow on all these surfaces by high cell viability	L929 fibroblasts and human Saos-2 osteoblasts	Bone	In vitro	[99]
PMMA-PCL-FA-GO	The viability of MG-63 osteoblast cells enhanced after the use of GO and FA in the PMMA-PCL polymer BC	MG-63 osteoblast cells	Bone	In vitro	[100]
PMMA-CS/GO (with 25 wt % CS/GO containing 2 and 0.3 wt % CS and GO respectively)	The improvement of cell viability, growth, and cell adhesion	Human osteosarcoma cell line MG-63	Bone	In vitro	[101]
PMMA/AG	The BC-AG has offered very conducive microenvironment to the surrounding cells for proper growth and proliferation to rapid mineral secretion	-	Bone	In vivo	[104]
PMMA/G (0.1 wt % G)	Did not invoke a cytotoxic response, thereby demonstrating an adequate level of biocompatibility	Osteoblast precursor cell line (MC3-T3)	Bone	In vitro	[105]
PMMA/GO (0.1 wt % GO)	Did not invoke a cytotoxic response, thereby demonstrating an adequate level of biocompatibility	Osteoblast precursor cell line (MC3-T3)	Bone	In vitro	[105]

**Table 4:** Characteristics and antibacterial behavior of CNTs and GO-based materials.

CNTs and GO Based Materials Features					Antibacterial Evaluation			
Type	Material	Fabrication Method	Concentration (µg/mL)	Bacterial Strains	Incubation Time (h)	Method	Inhibition	Ref.
Nanowalls	GO	Hummers and Offeman	-	*E. coli*	24	PC	59%	[110]
				*S. aureus*			74%	
Nanosheets	GO	Hummers and Offeman	175	*P. aeruginosa*	2	PC	100%	[111]
Nanopowder	GO	Hummers and Offeman	40	*E. coli*	2	PC	69.30%	[113]
Nanosheets	GO	Hummers and Offeman	100	*E. coli*	2	PC	17%	[114]
Nanosheets	GO	Hummers and Offeman	100	*E.coli*	3	PC	100%	[115]
			125	*S.iniae*				
Nanosheets	GO	Hummers and Offeman	85	*E. coli*	2	PC	98.50%	[116]
Nanosheets	GO	Hummers and Offeman	40	*E. coli*	2	PC	97.70%	[117]
Nanosheets	GO	Modified Hummers’procedure	100	*E. coli*	2.5	TEM	-	[118]
SWCNTs	CNTs	Electric arc discharge	100	*P. aeruginosa* and *S. aureus*	24	PC	50–60% and 70%	[119]
DWCNTs	CNTs	Catalytic Chemical Vapour Deposition						
MWCNTs	CNTs	Catalyst-assisted chemical vapor deposition						
SWCNTs	CNTs	CO disproportionation	5	*E. coli*	1	PC	86.80%	[120]
SWCNTs	CNTs	CO incorporated MCM-41	5	*E. coli*	1	PC	80.10%	[121]
MWCNTs	CNTs	Chemical Vapour Deposition method	5	*E. coli*	1	PC	24.40%	
SWCNTs	CNTs	CO decomposition	1/70 CNT/polymer(PLGA)	*S. epidermidis*	0.5	PC	98%	[122]
MWCNTs	CNTs	NanoLab productions	500	*S.typhimurium*, *B. subtilis*, *S. aureus*	1	PC	minor	[123]
SWCNTs	CNTs	NanoLab productions	200–250				−7 log	

PC: Plate Count; TEM: transmission electron microscopy.

## Abbreviations

ABCs	Acrylic bone cements	GO	Graphene oxide
BCs	Bone cements	HA	Hydroxyapatite
BPO	Benzoyl peroxide	HOb	Human osteoblast
CBNs	Carbon-based nanomaterials	MMA	Methyl methacrylate
CNTs	Carbon nanotubes	Mon	Monticellite
CS	Chitosan	MPS	[3-(Methacryloyloxy)propyl]trimethoxysilane
Ca-P	Calcium phosphate	MWCNTs	Multi-walled carbon nanotubes
DMA	Dynamic mechanical analysis	PCL	Polycaprolactone
DmpT	N,N-dimethyl-p-toluidine	PMMA	Polymethyl methacrylate
ECM	Extra-cellular matrix	rBMSC	Rat bone marrow mesenchymal stem cell
FA	Fluorapatite	RIC	Radical initiator concentration
f-CNT	Functionalized carbon nanotube	SIF	Stress intensity factor
FTIR	Fourier transform infrared spectroscopy	SWCNTs	Single-walled carbon nanotubes
FBT	Fractured bone treatments	TJR	Total joint replacement
G	Graphene	TNI	Thermal necrosis index
